# Investigating the Relationship between Magnesium levels and Diabetes Mellitus in Pregnant Women

**DOI:** 10.22088/IJMCM.BUMS.8.3.223

**Published:** 2019

**Authors:** Hadis Musavi, Fariba Mohammadi Tahroodi, Farzaneh Fesahat, Zinatossadat Bouzari, Seddigheh Esmaeilzadeh, Fatemeh Elmi, Shahla Yazdani, Zoleika Moazezi

**Affiliations:** 1 Student Research Committee, Babol University of Medical Sciences, Babol, Iran.; 2 Department of Biochemistry, School of Medicine, Kerman University of Medical Sciences, Kerman, Iran.; 3 Reproductive Immunology Research Center, Shahid Sadoghi University of Medical Sciences, Yazd, Iran.; 4 Infertility and Reproductive Health Research Center, Health Research Institute, Babol University of Medical Sciences, Babol, Iran.; 5 Cellular and Molecular Biology Research Center, Health Research Institute, Babol University of Medical Sciences, Babol, Iran.; 6 Department of Obstetrics and Gynecology, School of Medicine, Infertility and Reproductive Health Research Center Fatima Al-Zahra Research Center for Fertility and Infertility, Babol University of Medical Sciences, Babol, Iran.; 7 Student Research Committee, Babol University of Medical Sciences, Babol, Iran.; 8 Clinical Research Development Unit of Rouhani Hospital, Department of Internal Medicine, Babol University of Medical Sciences, Babol, Iran.; 9 Department of Endocrinology, Ayatollah Rouhani Hospital, Babol University of Medical Sciences, Babol. Iran.; # The first two authors have an equal contribution.

**Keywords:** Gestational diabetes mellitus, magnesium, serum albumin, diabetes mellitus, pregnancy

## Abstract

Gestational diabetes mellitus (GDM) is defined as one of the three main types of diabetes mellitus (DM). It is established that GDM is associated with exceeding nutrient losses owing to glycosuria. Magnesium (Mg), as one of the essential micronutrients for fetus development, acts as the main cofactor in most enzymatic processes. The aim of this study was to measure serum and cellular levels of Mg, albumin, creatinine, and total protein to further clarify the relationship between these components and DM in pregnant women. Blood samples were obtained from 387 pregnant women. The participants were classified into four groups based on their type of diabetes, namely GDM (n=96), DM (n=44), at high-risk of DM (n=122), and healthy controls (n=125). All participants' fasting blood sugar (FBS), creatinine, albumin, Mg, and total protein in the serum levels and red blood cell Mg (RBC-Mg) were measured during 24-28 weeks of gestation. Groups were compared for possible association between DM and abortion, gravidity, and parity. The serum levels of creatinine, FBS, albumin, Mg, and RBC-Mg were statistically different among four groups (P =0.001). Significant lower levels of RBC- Mg was observed in all studied groups in comparison with controls. Given a positive correlation between DM and abortion, it seems that decreased levels of RBC-Mg and serum albumin can increase the risk of abortion in pregnant women. Our data demonstrated significant alterations in albumin, Mg, and creatinine concentrations in women with DM or those at high risk of DM during their gestational age. It seems that the measurement of these biochemical parameters might be helpful for preventing the complications, and improving pregnancy outcomes complicated with DM.

Magnesium (Mg) is the second most abundant cation after potassium inside living cells. Out of 21-28 g Mg that is present in an adult's body, 99% is present in intracellular space and only 1% is present in extracellular fluid (1). In healthy individuals, plasma Mg concentrations are relatively stable within the range of 0.70-1.00 mM (2). Mg, as one of the essential micronutrients for fetus development, can act as the main cofactor in most enzymatic processes (3). On the other hand, Mg plays a key role in calcium homeostasis, affecting the activity of ATPase (4). Since calcium has an important role in insulin release and glucose metabolism, it can be concluded that this cation may play a prominent role in the processes of glucose and oxygen supply for cellular glucose oxidation (5). Determination of a threshold for Mg insufficiency status is usually difficult since it depends on the health status and other characteristics of the target population (6). Mg deficiency has been associated with increased risk of cardiovascular disease (7), type 2 diabetes (8), metabolic disorders (9), and pregnancy (10).

Gestational diabetes mellitus (GDM) is defined as one of the three main types of diabetes mellitus (DM) (i.e. type 1, type 2, and GDM), which is detected in almost 3-5% of pregnancies (11). GDM refers to carbohydrate intolerance causing hyperglycemia with the onset or first recognition during pregnancy (12). In addition, it is established that GDM is associated with exceeding nutrient losses owing to glycosuria (13). Bardicef et al. reported the reduction of intracellular free Mg in pregnant women affected with GDM. They also concluded that hypomagnesemia was involved in the occurrence of macrovascular complications during pregnancy (14). However, the role of Mg in DM and DM-induced vascular complications still remains unclear. Being aware of the role of Mg as an important factor in glucose metabolism can be helpful in prognosis of women who are at high risk of GDM. The aim of this study was to measure serum and cellular levels of Mg and other serum components including albumin, creatinine, and total protein, to further clarify the relationship between this cation and DM in pregnant women.

## Patients and methods

**Patients selection**

This study was conducted on 387 pregnant women with gestational age of 24 to 32 weeks who were referred to Ayatollah Rohani Hospital affiliated to Babol University of Medical Sciences during 2015-2016. The randomly selected pregnant women were divided into four groups according to their type of diabetes. The groups included pregnant women with GDM (group 1, N=96), pregnant women with apparent diabetes (group 2, N=44), high-risk pregnant women, including women with a history of GDM and obesity, GDM in their first-degree relatives, glycosmis or macrosomia infant (group 3, N=122) (15), and healthy pregnant women without a history of diabetes who were categorized as control group (group 4, N=125). Participants with a history of heart disease, gastrointestinal disorders, renal dysfunction, or hypertension were excluded. All participants filled out a questionnaire providing information about their pregnancy, demographic data, and history of any disorder (diabetes, chronic diseases, abortion, pregnancy, or high-risk child birth) either in them or in their family members. The use of all samples was approved by the Ethics Committee of Babol medical university with ethical number MuBABOL.HRI.REC.1395.56. All participants signed the informed consent for this research.

**Biochemical analyzes**

All participants underwent fasting blood sugar (FBS), creatinine, albumin, Mg, total protein levels in the serum, and red blood cell Mg (RBC-Mg) measurements during 24-28 weeks of gestation. Patients’ albumin, total protein, and creatinine blood levels were measured by Biuret test, Bromcresol Green method, and Jaffe method, respectively. Serum Mg level was measured by COBAS device (Switzerland) and RBC-Mg concentration by BIOLABO reagents kit (France). Pregnant women without a history of diabetes underwent a glucose challenge test (GCT) (50 g of glucose) during 24-28 weeks of gestation. One hour later, blood samples were re-collected from all participants to measure blood glucose. Glucose concentrations ≥130 mg/dl in GCT were considered to be a positive test. After a week and following a 3-day diet containing 150 g of carbohydrate, oral glucose tolerance test (OGTT) was performed using 100 g glucose for women with GCT positive test. Carpenter and Coustan diagnostic thresholds (95 mg/dL, 180 mg/dL, 155 mg/dL, and 140 mg/dL plasma glucose values for fasting, 1-h, 2-h, and 3-h after 100 g OGTT) were used for the interpretation and diagnosis of GDM (16). 

**Statistical analysis**

The data are presented as mean ± SD, median and percentage. Either the independent samples t test or one-way ANOVA test was used to discover any differences among four groups. All analyses were performed using PASW statistics 18 (SPSS). Based on One Sample Kolmogorov- Smirnov test, our data did not have a normal distribution. Therefore, the comparisons between two groups were performed by non-parametric test. Pearson's correlation was run to calculate correlations. Logistic regression was also applied to examine the relationship between the measured factors and the rate of fetus/embryo abortion. Chi-square and Fisher exact test were used to determine the relationship between DM and gravidity or abortion across four groups. Moreover, a receiver- operating characteristic (ROC) curve was performed for evaluating diagnostic performance and accuracy of a test to discriminate between cases and controls by ROC curve in MedCalc statistical software. A value <0.05 was considered statistically significant. The Tukey test was performed to test all pairwise comparisons among means.

## Results

In this study, participants of four groups were similar in terms of age (P =0.8), height (P =0.9), and BMI in different time intervals during pregnancy (P>0.05) (Table 1). Table 1 shows that serum levels of creatinine, FBS, albumin, Mg, and RBC- Mg were statistically different among four groups (P =0.001). Despite significant lower levels of RBC-Mg in experimental groups (1, 2 and, 3) compared to those in controls (group 4), no significant difference between GDM pregnant women (group 1) and controls was observed concerning serum levels of Mg (P =0.9).

Based on observed data, there was a significant negative correlation between FBS and serum Mg as well as RBC- Mg concentration. On the other hand, we found a significant positive correlation between Mg levels and albumin, creatinine, and ratio of urine albumin to total protein (Table 2).

Logistic regression analysis showed decreased levels of RBC-Mg with an odds ratio (OR) of 2.9 (P=0.02). Additionally, it was found that serum albumin (OR=0.3, P=0.02) could increase the risk of abortion in pregnant women. However, we did not observe any significant relationship between the rate of abortion in pregnant women and serum Mg, and other variables concentrations (P>0.05).

**Fig. 1:**
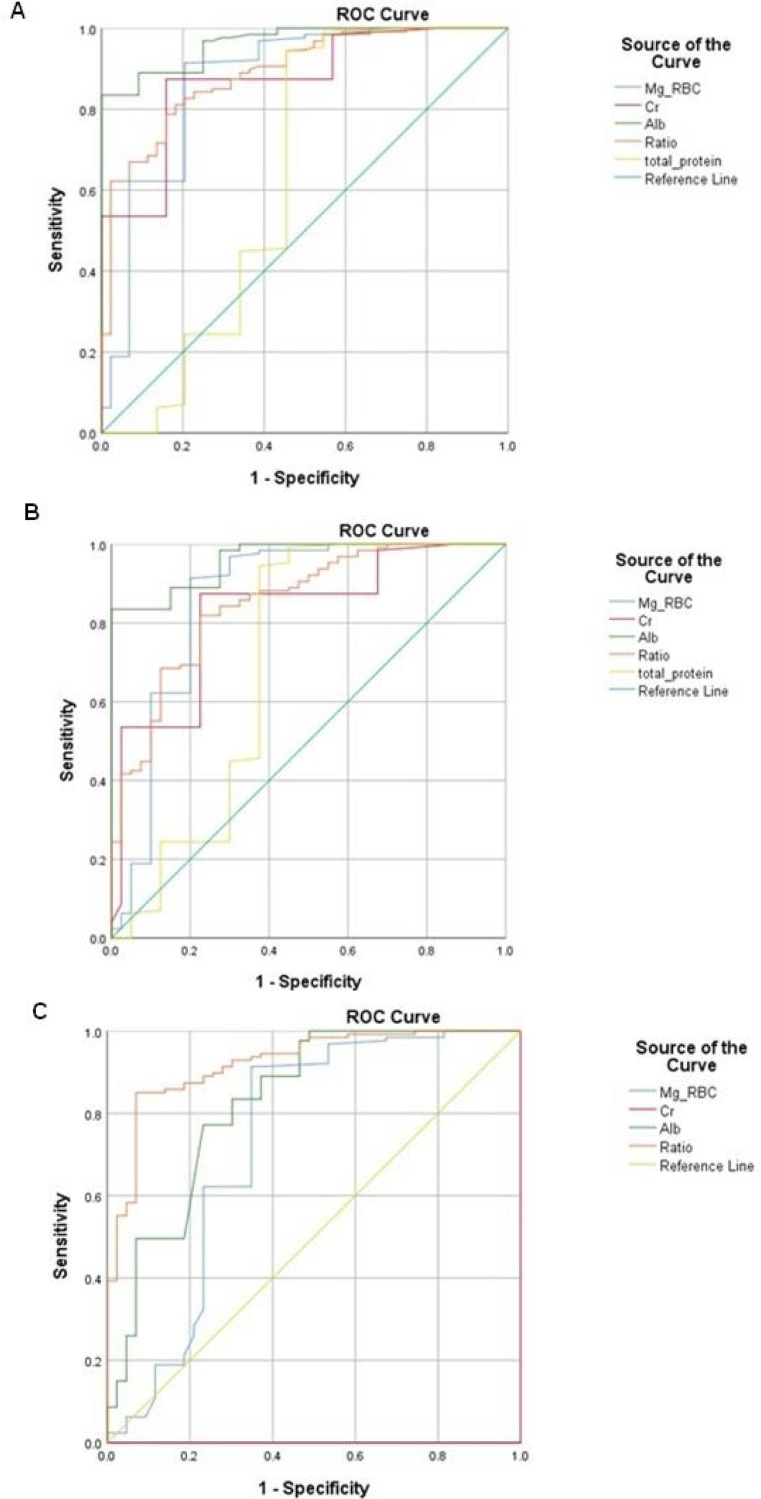
**ROC curve evaluating the specificity and sensitivity of all studied variables. **ROC: receiver-operating characteristic; AUC: area under the curve, Cr: creatinine; Mg-RBC: red blood cell- magnesium; ALB: albumin. Each ROC curve shows the values of gestational diabetic (GDM) pregnant women (group 1), diabetic pregnant woman (group 2), high risk patient for diabetes (group 3) in comparison with controls (group 4). Panel A: (1-4); Panel B: (2-4); Panel C: (3-4)

Chi-square test revealed no correlation between gravity and DM (P=0.06). However, there was a positive correlation between DM and increased parity as well as fetus abortion (P =0.001) (Table 3). According to chi coefficient, the severity of this correlation was about 18 %. Moreover, high specificity and sensitivity with significant values were observed in the ROC curves (Figure 1 and Table 4). The best score belonged to RBC-Mg concentrations with a sensitivity of 92% and an accuracy of 82% in identifying patients with DM (type 1 and type 2). The area under the ROC curves for RBC-Mg levels was 80%.

**Table 1:** Comparison of mean serum levels of creatinine, fasting blood sugar, albumin, total protein, and Mg and demographic characteristics across four groups

**Variable**	**Groups**					**P-value**
	**1 (N=99)**	**2 (N=50)**	**3 (N=121)**	**4 (N=129)**	**CI ** ^¥^	
**Age (Year)**	26.58±4.9	27.09±5.9	26.34±5.3	26.4±6.01	25.9-27.08	0.8*
**Height (cm)**	160.9±7.2	160.56±7.2	160.56±7.2	160.4±6.5	159.9-61.3	0.9*
**Primary BMI(Kg/m** ^2^ **)**	27.85±4.8	22`8.37±4.9	27.86±5.2	26.5±4.5	27.01-27.9	0.07*
**24-28 weeks BMI (Kg/m** ^2^ **)**	30.1±5	29.63±5.8	29.8±5	29.4±4.3	29.3-30.3	0.4*
**Final BMI (Kg/m** ^2^ **)**	32.31±4.8	33.21±5.4	32.11±5.3	31.6±4.4	31.6-32.6	0.3*
**Serum creatinine (mg/dL)**	0.6±0.01	0.6±0.1	0.7±0.1	0.7±0.05	0.6-0.7	0.001* 0.001^a-e^ 0.7 ^f^
**FBS** **(mg/dL)**	92.95±4.47	220±52.8	93.6±4.5	85.35±9.4	100.6-109.7	0.001* 0.003^a^ 0.001^b-f^
**Serum albumin** **(mg/dL)**	3.4±0.09	3.4±0.1	3.4±0.2	3.9±0.2	3.5-3.6	0.001* 0.001^a,d,e^ 0.1^b^ 0.4^c^ 1^f^
**Total protein** **(mg/dL)**	6.2±0.34	6.1±0.27	7.1±5.3	6.3±0.09	6.2-6.8	0.64* 0.2^a-c^ 0.9^d-f^
**RBC magnesium** **(mg/dL)**	4.7±0.24	4.6±0.35	4.8±0.29	5.1±0.17	4.8-4.9	0.001* 0.001^a-e^ 0.03^f^
**Serum ** **magnesium** **(mg/dL )**	1.72±0.13	1.65±0.15	1.73±0.12	1.72±0.10	1.70-1.73	0.001* 0.9^a^ 0.004^c,e^ 0.001^b,d,f^
**Albumin**/**Total protein**	0.5±0.03	0.5±0.03	0.5±0.03	0.6±0.04	0.55-0.57	0.001* 0.001^a-e^ 0.7^f^

BMI: body mass index; FBS: fasting blood sugar; RBC: red blood cell; CI: confidence interval of 95%. 1: gestational diabetic pregnant women group, 2: diabetic pregnant woman group; 3: high-risk patient for diabetes;4: control group. Data are presented as mean±SD or median (lower bound, upper bound) ^¥^. *shows significant difference based on One-way ANOVA and Tukey-HSD tests. P-value between two groups were as follows: 1 vs. 4 ^a^, 2 vs. 4 ^b^, 3 vs. 4 ^c^, 1 vs. 2 ^d^, 1 vs. 3 ^e^, 2 vs. 3 ^f^.

**Table 2:** Correlations between variables among the studied groups

**Variable **	**Serum Creatinine**	**FBS**	**Albumin**	**Albumin/** **Total protein**	**Total protein**	**RBC magnesium**
**All groups (1-2-3-4)**						
**Serum** **Magnesium**	- -	-0.2 0.001	0.09 0.09	0.9 0.004	0.8 0.01	0.2 0.001
**RBC magnesium**	0.3 0.001	-0.3 0.001	0.5 0.001	0.3 0.001	0.9 0.06	- -
**Groups of 1- 4**						
**Serum ** **Magnesium**	0.9 0.001	-0.9 0.001	0.6 0.001	0.5 0.001	0.8 0.2	0.9 0.001
**RBC magnesium**	0.4 0.001	-0.3 0.001	0.5 0.001	0.4 0.001	0.9 0.1	- -
**Groups of 2-4**						
**Serum ** **Magnesium**	0.3 0.02	-0.3 0.001	0.1 0.08	0.2 0.09	0.1 0.1	0.2 0.04
**RBC magnesium**	0.3 0.001	-0.5 0.001	0.40.001	0.03 0.001	0.2 0.06	- -
**Groups of 2-4**						
**Serum ** **Magnesium**	0.1 0.2	-0.04 0.001	0.4 0.06	0.6 0.001	0.5 0.001	0.2 0.01
**RBC magnesium**	0.3 0.001	-0.2 0.001	0.4 0.001	0.3 0.02	0.5 0.001	- -

BMI: body mass index; FBS: fasting blood sugar; RBC: red blood cell. 1: gestational diabetic pregnant women group, 2: diabetic pregnant woman group; 3: high-risk patient for diabetes; 4: control group. The P-values <0.05 indicate statistical significance. The Pearson correlation test was used. In each cell, the top value is r and the bottom is the P-value.

**Table 3:** Frequencies of variables among the studied groups

**Variable**		**Groups**			
		**1 (N=96)**	**2 (N=44)**	**3 (N=122)**	**4 (N=125)**
**Gravidity **	Primi-gravida	43 (25.7)	17 (10.2)	46 (27.6)	61 (36.5)
	Multi-gravida	53 (24.1)	27 (12.3)	76 (34.5)	64 (29.1)
**Parity **	Primi, Nulli-para	74 (24.1)	37 (12.1)	89 (29)	107 (34.8)
	Multi-para	22 (27.5)	7 (8.7)	33 (41.3)	18 (22.5)
**Abortion **	No	80 (27.8)	35 (12.2)	17 (26.7)	96 (33.3)
	Yes	16 (16.3)	9 (9.2)	45 (45.9)	28 (28.6)

1: gestational diabetic pregnant women group, 2: diabetic pregnant woman group; 3: high-risk patient for diabetes; 4: control group. All statistics were performed using Crosstabs test. Data were presented as Number (%).

**Table 4:** Diagnostic performance and accuracy of variables between cases and controls

Variable *	**Sensitivity(%)**	**Specificity(%)**	**Cut off**	**P-value**	**AUC**
**1-4**					
**RBC-Magnesium**	92%	82%	4.9	0.001	0.8
**Creatinine**	88%	82%	0.7	0.001	0.7
**Magnesium**	80%	40%	1.6	0.04	1.6
**Albumin**	90%	83%	3.5	0.001	0.9
**Total protein**	83%	56%	6.2	0.04	0.6
**Albumin/** **Total protein**	81%	82%	0.5	0.01	0.6
**2-4**					
**RBC-Magnesium**	92%	82%	4.9	0.001	0.8
**Creatinine**	88%	78%	0.7	0.03	0.8
**Magnesium**	80%	46%	1.6	0.05	0.6
**Albumin**	84%	88%	3.6	0.01	0.9
**3-4**					
**RBC-Magnesium**	83%	76%	5	0.001	0.8
**Creatinine**	85%	79%	0.7	0.001	0.7
**Magnesium**	84%	85%	3.6	0.001	0.9
**Albumin**	85%	86%	0.57	0.001	0.9

AUC: area under the receiver-operating characteristic (ROC) curve; FBS: fasting blood sugar; RBC: red blood cell. 1: gestational diabetic pregnant women group, 2: diabetic pregnant woman group; 3: high-risk patient for diabetes; 4: control group. The P-values <0.05 indicate statistical significance. The ROC curve was used.

## Discussion

In this prospective study, serum and RBC-Mg concentrations as well as albumin, creatinine, and total protein were evaluated in patients with different types of DM (type 1, type 2, and GDM) as well as in healthy pregnant women. Serum Mg concentration of group 2 (type 2 DM) was significantly lower when compared with other groups (P<0.001). However, no significant difference was observed between GDM patients and controls in terms of serum Mg concentration. However, we significantly observed lower concentrations of RBC-Mg in groups 1, 2, and 3 in comparison with controls (P = 0.001). In line with our findings, Tasdemir et al. found no significant difference between healthy pregnant women and women with GDM with respect to serum Mg concentration (17). In contrast, Mishu et al. declared that low serum Mg concentration in Bangladeshi gestational diabetic mothers may be due to Mg depletion caused by osmotic diuresis and devious hormonal effects (10). This discrepancy in results can be due to the fact that we included participants who were at their third trimester of pregnancy but participants in Mishu et al.' study included women in both second and third trimester (10).

DM is an endocrine disorder that is associated with increased blood glucose concentration. The increase in the prevalence of this disease, as a global health problem, is anticipated in the next decade (11). Following DM, patient's blood glucose, insulin resistance, and insulin deficiency usually increase, resulting in Mg wasting and finally increase for the need of Mg (11). Current evidence indicates that Mg deficiency may elevate the risk of type 2 DM due to its negative effects on insulin sensitivity and glucose control (11, 18). It has been shown that one of the important factors in insulin resistance in DM patients is the low level of serum Mg (19). A recent report conducted by Bertinato et al. in Canada demonstrated that diabetes was the main predictor of serum Mg levels given that having each type of DM (type 1, type 2, or both) was associated with decreased serum Mg (18). Considering significant negative correlation between Mg levels and FBS in our study, it seems that increased FBS in DM patients may reduce both serum Mg and RBC-Mg concentration.

In the present study, we significantly detected lower concentrations of albumin and creatinine in GDM patients in comparison with controls. In addition, a significant positive correlation was observed between albumin concentration and RBC-Mg concentration in groups 1, 2 and, 3 but not in controls.

One of the most important factors in Mg homeostasis is albumin. Albumin can interfere with Mg concentration by preventing glomerular excretion of Mg due to its binding to Mg and its positive effect on Mg homeostasis (20). The decrease in ratio of urine albumin to total protein was associated with an increase in albumin renal excretion in GDM patients (21). Increase of glomerular filtration of albumin can lead to high amount of Mg, resulting in hypomagnesemia. In other words, along with decrease of serum albumin in women with GDM, hypomagnesemia and Mg reduction in RBCs can also occur (21).

Moreover, we observed a significant positive association between DM (either GDM or type 2) and increased parity (P =0.001). Akter et al. conducted a cross-sectional study indicating that multiparity or gravidity can be a risk factor for metabolic syndromes (22). Multi parity or gravidity may be a risk factor for metabolic syndrome.

Multi parity or gravidity may be a risk factor for metabolic syndrome.

In a prospective cohort study by Muelle et al., the association between parity and DM was evaluated in Singaporean Chinese women. The results showed a positive association between parity and DM risk (P <0.001) (23) that is in line with our findings. In other words, Muelle et al. concluded that increased parity may be a risk factor for DM in Chinese women (23).

The findings of a randomized clinical trial demonstrated that GMD therapy could diminish critical perinatal morbidity and enhance woman’s health-related quality of life (24). In addition, a large cohort study yielded a significant association between DM (type 2 and early GDM) and poor pregnancy outcomes in women who were diagnosed with DM at <12 weeks of gestation (25).

In conclusion our data demonstrated significant alterations in albumin, Mg, and creatinine concentrations in women with DM or those at high risk of DM during their gestational age. It seems that the measurement of these biochemical parameters might be helpful for preventing the complications, and improving pregnancy outcomes complicated with DM.
